# Cerebral blood flow velocity during simultaneous changes in mean arterial pressure and cardiac output in healthy volunteers

**DOI:** 10.1007/s00421-021-04693-6

**Published:** 2021-04-22

**Authors:** Sole Lindvåg Lie, Jonny Hisdal, Lars Øivind Høiseth

**Affiliations:** 1 Faculty of Medicine, University of Oslo, Oslo, Norway; 2 Section of Vascular Investigations, Department of Vascular Surgery, Oslo University Hospital, 0424 Oslo, Norway; 3 Department of Anesthesiology, Division of Emergencies and Critical Care, Oslo University Hospital, Oslo, Norway

**Keywords:** Blood pressure, Cardiac output, Cerebrovascular circulation, Hypovolemia, Volunteers

## Abstract

**Purpose:**

Cerebral blood flow (CBF) needs to be precisely controlled to maintain brain functions. While previously believed to be autoregulated and near constant over a wide blood pressure range, CBF is now understood as more pressure passive. However, there are still questions regarding the integrated nature of CBF regulation and more specifically the role of cardiac output. Our aim was, therefore, to explore the effects of MAP and cardiac output on CBF in a combined model of reduced preload and increased afterload.

**Method:**

16 healthy volunteers were exposed to combinations of different levels of simultaneous lower body negative pressure and isometric hand grip. We measured blood velocity in the middle cerebral artery (MCAV) and internal carotid artery (ICAV) by Doppler ultrasound, and cerebral oxygen saturation (ScO_2_) by near-infrared spectroscopy, as surrogates for CBF. The effect of changes in MAP and cardiac output on CBF was estimated with mixed multiple regression.

**Result:**

Both MAP and cardiac output had independent effects on MCAV, ICAV and ScO_2_. For ICAV and ScO_2_ there was also a statistically significant interaction effect between MAP and cardiac output. The estimated effect of a change of 10 mmHg in MAP on MCAV was 3.11 cm/s (95% CI 2.51–3.71, *P* < 0.001), and the effect of a change of 1 L/min in cardiac output was 3.41 cm/s (95% CI 2.82–4.00, *P* < 0.001).

**Conclusion:**

The present study indicates that during reductions in cardiac output, both MAP and cardiac output have independent effects on CBF.

**Supplementary Information:**

The online version contains supplementary material available at 10.1007/s00421-021-04693-6.

## Introduction

The brain has a high metabolic rate and limited substrate stores, making regulation of cerebral blood flow (CBF) essential to maintain its functions (Meng et al. 2015; Willie et al. 2014). The main regulators of CBF are believed to be the arterial partial pressure of CO_2_ (PaCO_2_), mean arterial pressure (MAP), cerebral metabolism and autonomic nervous system (Willie et al. 2014). Even though still highly influential, Lassen’s presentation of cerebral autoregulation (CA) (Lassen 1959) has been criticized, as the within subject autoregulatory blood pressure range seems to be narrower than previously stated. In addition, the relationship between MAP and CBF seems to be more pressure passive (Numan et al. 2014).

MAP is the product of systemic vascular resistance (SVR) and cardiac output (disregarding right atrial pressure), and one value of MAP can be seen with different combinations of these two factors. Maintaining constant CBF during a reduction in cardiac output would require extracerebral vascular resistance to increase disproportionally to that of the brain, in accordance with the “selfish brain hypothesis” (Hart 2016). However, there is a convincing amount of evidence pointing to a significant effect of cardiac output on CBF, both in disease and in health (Neumann et al. 2019). In healthy volunteers, several studies have reported reduced cerebral blood velocity with reduced cardiac output during simulated hypovolemia such as lower body negative pressure (LBNP) (Zhang and Levine 2007; Tymko et al. 2016; Levine et al. 1994; Ogoh et al. 2005). There seems to be an effect of cardiac output independent of MAP (Ogoh et al. 2005; Meng et al. 2015), and the notion of strict autoregulation has largely been abandoned (Willie et al. 2014).

There may be redundancy in the different mechanisms affecting CBF, and they may use the same vascular effectors. There is a need for studies integrating different stimuli affecting CBF to further elucidate their effects and interactions. We therefore exposed subjects to isometric hand grip (IHG) during different levels of LBNP. LBNP is a model of central hypovolemia, reduced preload and thereby cardiac output as blood is redistributed from the upper to the lower body (Goswami et al. 2019). IHG increases muscle and skin sympathetic nervous activity and thereby MAP and cardiac afterload with sustained submaximal isometric muscle contraction (Seals 1993).

The aim of the present study was to explore the effects and interactions of changes in MAP and cardiac output on CBF by performing simultaneous LBNP and IHG while measuring middle cerebral artery (MCA) blood velocity (MCAV), internal carotid artery (ICA) blood velocity (ICAV) and cerebral oxygen saturation (ScO_2_). We hypothesized that there would be significant associations both between changes in MAP and CBF, and between changes in cardiac output and CBF.

## Methods

### Subjects

After regional ethics committee approval (REC South East A, ref. 2017/136) and written informed consent, 16 healthy volunteers > 18 years were included in the study. Exclusion criteria were pregnancy, any condition limiting physical performance or requiring regular medication (contraceptives exempted) or history of any syncope (presumed vasovagal syncope with known precipitating factor exempted). Subjects refrained from caffeine and strenuous physical activity on the day of the experiment. All experiments were performed between 8 a.m. and 4 p.m. The subjects were accustomed with the setup and acclimatized for 20–30 min before the start of the experiment.

### Experimental design

**Fig. 1:**
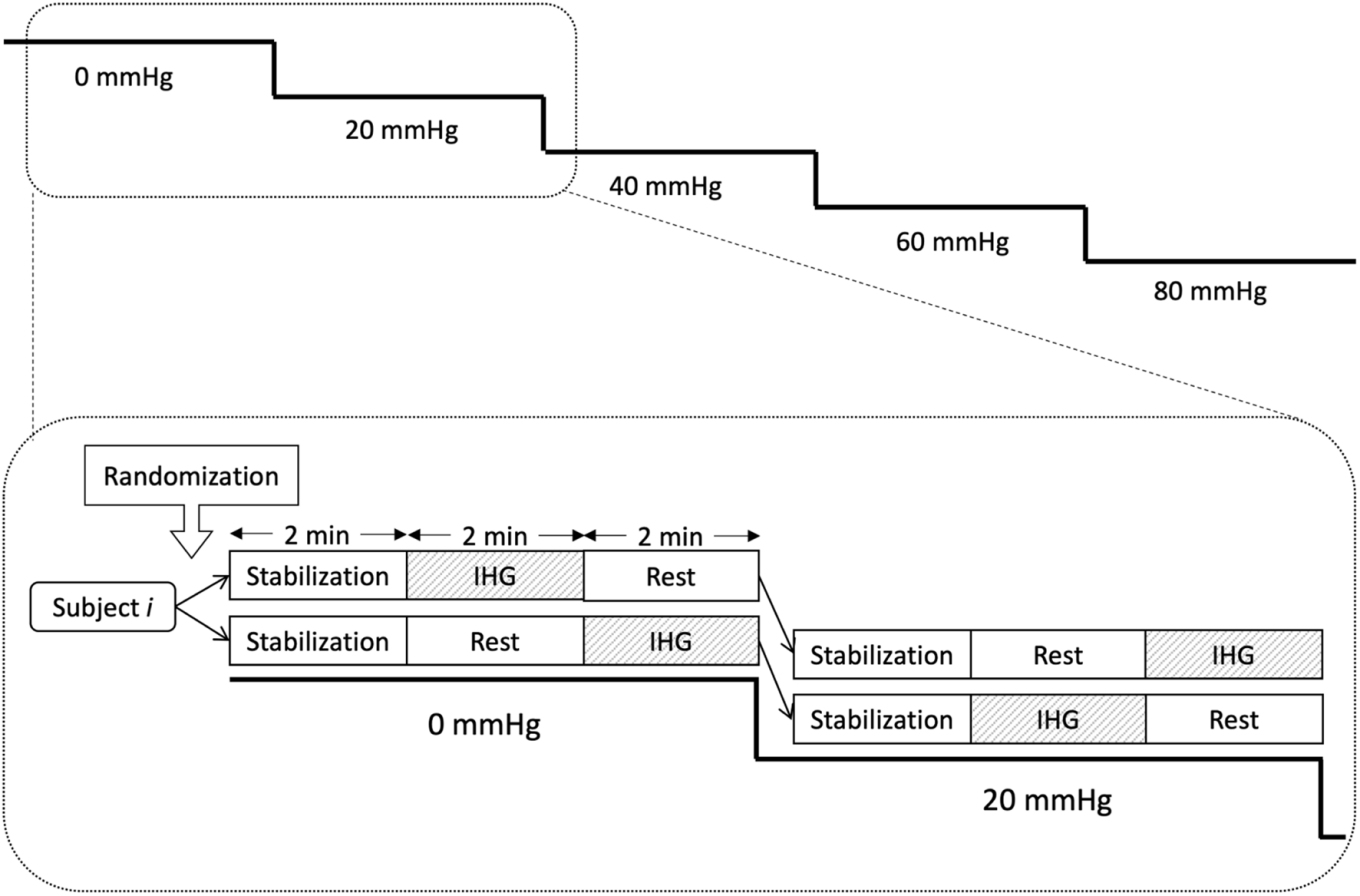
Study protocol. All lower body negative pressure (LBNP) levels lasted for 6 min, with 2 min of stabilization, 2 min of rest and 2 min of isometric hand grip (IHG). Subjects were randomized to start with IHG or rest at LBNP 0, and the order of rest/IHG on the following LBNP levels was alternated

The subjects were exposed to stepwise LBNP of 0, 20, 40, 60 and 80 mmHg. Each LBNP level lasted 6 min and started with 2 min stabilization. Thereafter, the subjects were exposed to 2 min of IHG and 2 min of rest. The subjects were randomized to start with IHG or rest at LBNP 0, with alternating order at subsequent LBNP levels (Fig. 1). To get a balanced design, the subjects were block-randomized with block sizes 2 or 4, using the “blockrand” package in R (Snow). LBNP was relieved before completing LBNP 80 if the subjects displayed signs or symptoms of impending circulatory collapse such as a sudden drop in heart rate or blood pressure, light headedness or nausea. Only completed LBNP levels were used in the analysis, i.e. the LBNP level at which decompensation occurred was removed from the analysis.

### Interventions and measurements

The subjects were placed in the LBNP chamber (Hisdal et al. 2003) sealed just above the iliac crest. A three-lead ECG was obtained using Bio Amp/ PowerLab (ADInstruments, Bella Vista, Australia) and exported to Lab Chart 8.1.9 (ADInstruments) for calculation of heart rate (HR). Arterial blood pressure waveform was measured with the volume-clamp method on the third finger of the left hand (Nexfin; BMEYE, Amsterdam, The Netherlands) and exported to LabChart where mean arterial pressure (MAP) was calculated as the time-weighted integral. Cardiac stroke volume was measured continuously with suprasternal Doppler (SD-50; Vingmed Ultrasound, Horten, Norway) (Eriksen and Walløe 1990). Velocity–time integrals for each heartbeat were calculated in LabChart and multiplied by left ventricular outflow tract area from echocardiographic measurements obtained in parasternal long-axis to obtain stroke volume. Cardiac output was calculated as the product of stroke volume and heart rate. End-tidal CO_2_ (ETCO_2_) was measured by sidestream capnography (Cap10; Medlab medizinische Diagnosegeräte GmbH, Stutensee, Germany), sampled from a loose-fitting facemask offering minimal resistance and added dead-space. Cerebral oxygen saturation (ScO_2_) was measured using near-infrared spectroscopy (NIRS; Invos 5100C cerebral/somatic oximeter; Somanetics, Troy, MI, USA). Sensors were placed on the left and right forehead and averaged. Data were extracted from the serial output every 7–8 s to software in LabVIEW 14.0 (National Instruments, Austin, TX, USA).

### Middle cerebral artery blood velocity

Middle cerebral artery blood velocity (MCAV) was measured using triplex ultrasound (Logiq S8; GE Healthcare, Boston, MA, USA) with a 2 MHz probe over the right temporal window by the same experienced operator. The MCA was identified, and a pulsed Doppler sample volume was placed aiming for a clearly delineated Doppler signal with the lowest possible angle of insonation. The sample volume was not moved through the experiment. The recordings were exported as.avi-files to Brachial Analyzer 6.4.7 (Medical Imaging Application, Coralville, IA, USA) where MCAV was calculated by automatic tracing of the Doppler waveform.

### Internal carotid artery blood velocity, diameter and flow

Internal carotid artery blood velocity (ICAV) and diameter was measured using duplex ultrasound (Vivid E9; GE Vingmed, Horten, Norway) with a 9 MHz linear probe by the same experienced operator. ICA was identified by the location of the carotid bifurcation in B-mode and the morphology of the Doppler waveform. A pulsed Doppler sample volume was placed 1.5–2 cm distal to the carotid bifurcation and adjusted for the angle of insonation, which was < 60°. The sample volume was placed in the middle of the vessel and not moved through the experiment (Thomas et al. 2015). The recordings were exported as DICOM-files to EchoPAC 202 (GE Vingmed) where ICAV was calculated by automatic tracing of the Doppler waveform.

### Isometric hand grip

IHG was performed by gripping the right hand on a handle displaying the applied force to the subject. Before the experiment, we measured the maximum voluntary contraction force as the average of three repeated attempts. The subjects were instructed to keep 40% of this force during the IHG by reading the display.

### Data processing

All analog signals were sampled in LabChart at 1000 Hz. Data for each heartbeat were defined by the ECG RR-interval and exported as.txt-files and handled in R 3.6.1 (R Foundation for Statistical Computing, Vienna, Austria)/ RStudio 1.1.442 (RStudio, Boston, MA, USA) using the “tidyverse” packages (Wickham 2017). Data for MCAV and ICAV were synchronized with beat-by-beat data from LabChart in R/ RStudio. Mean values for periods of 30 s were calculated, trimming the 5% highest and lowest values to remove noise generated by e.g. motion or extrasystoles.

The ultrasound machines only allowed for maximum 4 min continuous recordings. To be able to capture the transition phase with release of IHG in all subjects, we were not able to record both the whole IHG and “rest” sequences. We did therefore not record the first minute of the “rest” sequence. The final analyses included the last minute of the “rest” sequence (two values each of 30 s averages) and the whole 2 min IHG-sequence (four values). As the release of IHG was accompanied by very rapid hemodynamic changes, the transition phases with release of IHG are not included in the present analyses. ICA diameter was measured manually at end-diastole and end-systole 15 s into each 30 s sequence. ICA mean diameter for cross-sectional area estimation was calculated as [(systolic diameter × 1/3) + (diastolic diameter × 2/3)]. ICA blood flow was calculated as [peak envelope velocity/2 × *π* × (mean diameter/2)^2^ × 60].

### Statistics

Based on previous data, simulations in a linear mixed regression model showed that 12 subjects would detect a Pearson correlation coefficient of 0.4 between blood velocity and MAP with *α* = 0.05 and (1 − *β*) = 0.9, assuming all subjects completing all LBNP levels. Because some subjects were assumed to decompensate before completing all LBNP levels, we included 16 subjects.

Associations between the CBF surrogates and the independent variables were examined using a linear mixed effects model with subjects as a random effect using the “lme” function of the “nlme” package in R (Pinheiro et al. 2017). The independent variables were entered as fixed effects. For every CBF surrogate, linear mixed effects analyses were conducted for each independent variable to ensure statistically significant effects before entering them in a multivariable model with an interaction effect. The interaction effect was included to explore possible dependencies between MAP and cardiac output, and removed from the final model unless statistically significant. Model assumptions were checked using histograms, QQ-plots and by plotting standardized residuals vs. fitted values. When LBNP level was entered as a factor, levels were compared using the “glht” function of the “multcomp” package in R (Hothorn et al. 2008) with “single-step” correction for multiple comparisons. Values are mean (SD) unless otherwise stated. *P* < 0.05 was considered statistically significant. Precision was calculated as 1.96 × √(residual mean square) in a one-way ANOVA with subjects as factors (Bland and Altman 1999).

## Results

**Fig. 2:**
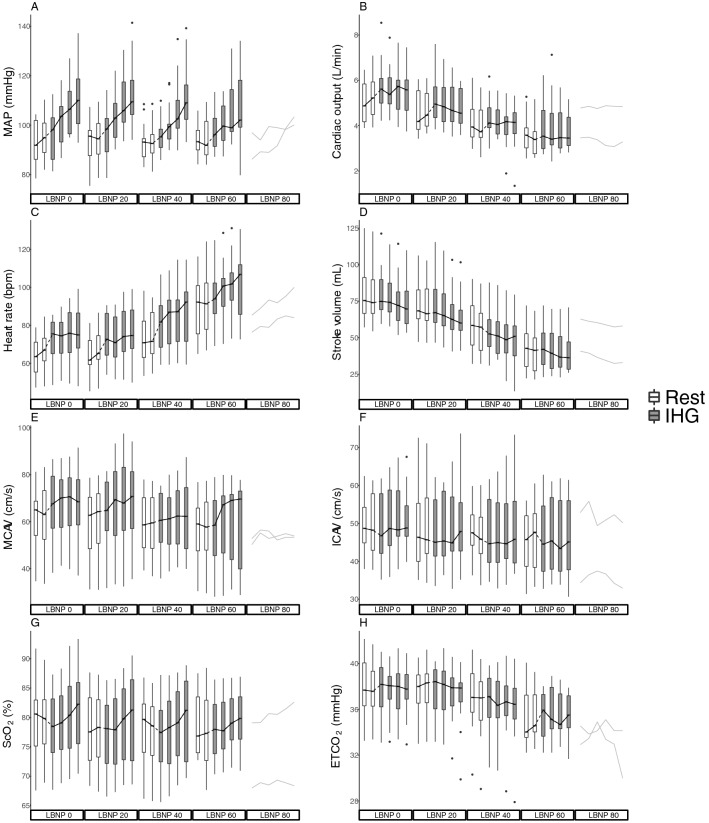
**a** Mean arterial pressure (MAP), **b** cardiac output, **c** heart rate, **d** stroke volume, **e** middle cerebral artery velocity (MCAV), **f** internal carotid artery velocity (ICAV), **g** cerebral oxygen saturation (ScO_2_) and **h** end-tidal carbon dioxide (ETCO_2_) on all lower body negative pressure (LBNP) levels. At LBNP 80, data from the two subjects completing the level are displayed. Boxes are median and 25th and 75th percentiles. Whiskers are × 1.5 interquartile range. Black dots are outliers. White boxes represent rest periods and gray boxes are isometric hand grip (IHG) periods. Of note, at every other LBNP level, alternating within subject, IHG was performed before rest

16 subjects (9 female) with age 24 (3) years, height 177 (11) cm, weight 71 (14) kg and body mass index 23 (3) kg/m^2^ were included. All subjects completed LBNP 0 and LBNP 20, 15 subjects completed LBNP 40, 11 subjects completed LBNP 60 and two subjects completed LBNP 80. MAP, cardiac output, heart rate, stroke volume, MCAV, ICAV, ScO_2_ and ETCO_2_ through the experiment are presented in Fig. 2 and shows the intended increase in MAP with IHG, and reduction in cardiac output with LBNP. The precision of measurement was 4.37 cm/s for MCAV, 4.20 cm/s for ICAV and 0.022 cm for ICA diameter.

### Effects on MCAV

#### Bivariable analyses

**Fig. 3:**
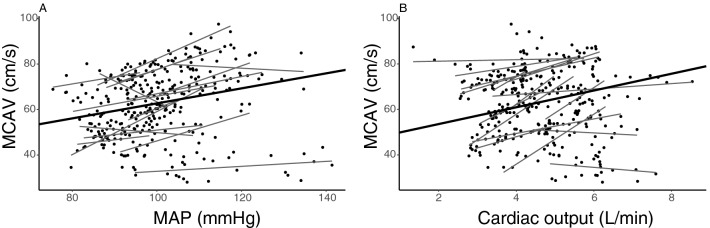
Middle cerebral artery velocity (MCAV) regressed on **a** mean arterial pressure (MAP) and **b** cardiac output. The black line represents the mixed linear regression analysis, and black dots represent subject values with corresponding simple linear regressions represented as gray lines

The associations between MAP and MCAV, and cardiac output and MCAV, are presented in Fig. 3. In the regression analyses, both MAP and cardiac output were significantly associated with MCAV. The estimated effect of a change of 10 mmHg in MAP on MCAV was 3.43 cm/s (95% CI 2.72–4.13; *P* < 0.001) (Fig. 3, panel A and Appendix, Regression 1). The estimated effect of a change of 1 L/min in cardiac output on MCAV was 3.69 cm/s (95% CI 3.02–4.36; *P* < 0.001) (Fig. 3, panel B and Appendix, Regression 2).

Exploration of a possible non-linear relation between MCAV and MAP was explored with polynomial regression, without achieving better model-fit than assuming a linear relationship between MCAV and MAP (Appendix, Regression 20).

#### Multivariable analyses

When entering both MAP and cardiac output in a multivariable model, an interaction term was not statistically significant (*P* = 0.067) and removed. The effects of MAP and cardiac output were both significant, with a 10 mmHg change in MAP corresponding to a change in MCAV of 3.11 cm/s (95% CI 2.51–3.71; *P* < 0.001), and a 1 L/min change in cardiac output corresponding to a change in MCAV of 3.41 cm/s (95% CI 2.82–4.00; *P* < 0.001) (Appendix, Regression 3).

**Fig. 4:**
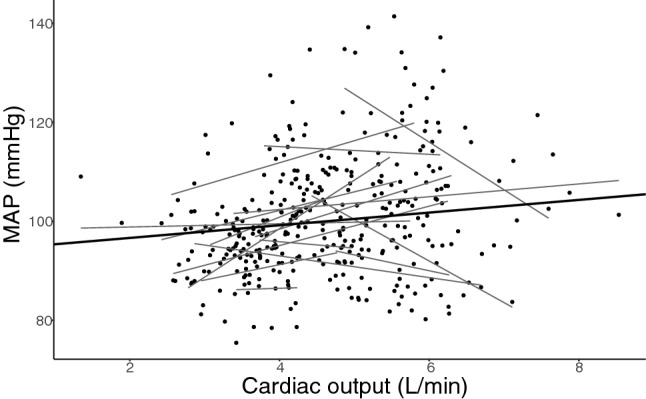
Mean arterial pressure (MAP) regressed on cardiac output. The black line represents the mixed linear regression analysis, and black dots represent subject values with corresponding simple linear regressions represented as gray lines

Although MAP and cardiac output did not seem strongly correlated (Fig. 4), they are mathematically coupled (Archie 1981) through the equation MAP ~ cardiac output × SVR (disregarding central venous pressure). We therefore entered LBNP level as a factor as a proxy for cardiac output in the model, giving significant main effects of both LBNP level and MAP (no interaction effects were statistically significant) (Appendix, Regression 4, Fig. A). In this model, a change of 10 mmHg in MAP corresponded to a change in MCAV of 2.89 cm/s (95% CI 2.35–3.44; *P* < 0.001).

### Effects on ICAV

#### Bivariable analyses

The associations between MAP and ICAV, and cardiac output and ICAV, are presented in Appendix, Regressions 8 and 9, Figs. D and E. The estimated effect of a 10 mmHg change in MAP on ICAV was 0.88 cm/s (95% CI 0.29–1.46; *P* = 0.004). The estimated effect of a 1 L/min change in cardiac output on ICAV was 0.83 cm/s (95% CI 0.21–1.44; *P* = 0.009).

#### Multivariable analyses

When entering both MAP and cardiac output in a multivariable model, both the main effects and an interaction term were statistically significant. The interaction effect indicated a smaller effect of MAP as cardiac output increased, and vice versa. The results of these regression analyses and estimated effects of each predictor at the 1st, 2nd and 3rd quartile of the other are presented in Appendix, Regression 10.

### ICA blood flow

The associations between MAP and ICA blood flow, and cardiac output and ICA blood flow, are presented in Appendix, Regressions 12 and 13, Figs. G and H. ICA diameters were measured in 14 subjects. When entering cardiac output and MAP as explanatory variables in a bivariable analysis, there was a marginal, but statistically significant effect of cardiac output on ICA blood flow. The estimated effect of a change of 1 L/min in cardiac output on ICA blood flow was 11.94 mL/min (95% CI 4.54–19.33, *P* = 0.002). There was no significant effect of MAP on ICA blood flow. The effects of MAP and cardiac output were similar when entered in a multivariable analysis (Appendix, Regressions 14).

### Effects on ScO_2_

#### Bivariable analyses

The associations between MAP and ScO_2_, and cardiac output and ScO_2_, are presented in Appendix, Regressions 15 and 16, Figs. I and J. The estimated effect of a change of 10 mmHg in MAP on ScO_2_ was 1.02% (95% CI 0.76–1.27; *P* < 0.001). The estimated effect of a change of 1 L/min in cardiac output on ScO_2_ was 0.64% (95% CI 0.34–0.90; *P* < 0.001).

#### Multivariable analyses

When entering both MAP and cardiac output in a multivariable model, both the main effects and an interaction term were statistically significant. The interaction effect indicated a smaller effect of MAP as cardiac output increased, and vice versa. The results of these regression analyses and estimated effects of each predictor at the 1st, 2nd and 3rd quartile of the other are presented in Appendix, Regression 17.

### MCAV, ICAV and ScO_2_

MCAV, ICAV and ScO_2_ were all statistically significantly associated with each other, as presented in Appendix (Regressions 11, 18 and 19, and Figs. F, K and L).

### ETCO_2_

ETCO_2_ through the experiment is presented in Fig. 2, panel H, and was associated with cardiac output (Appendix, Regression 6 and Fig. C). When entering ETCO_2_ as an explanatory variable in a multivariable analysis with MAP and cardiac output, there was a statistically significant effect of ETCO_2_. Also, in this model the effect of cardiac output on MCAV was reduced, but the effect of MAP was practically unaffected (Appendix, Regression 7).

## Discussion

The main finding of the present study was that during reductions in cardiac output, both MAP and cardiac output had independent effects on MCAV. Similar effects were found for MAP and cardiac output on ICAV and ScO_2_.

### Autoregulation

Cerebral autoregulation (CA) has long been presented in textbooks as a mechanism to maintain constant CBF despite changes in MAP (Lassen 1959; Numan et al. 2014). This is theoretically compatible with Poiseuille’s law of flow but may in some circumstances require cerebral vascular resistance to act uncoupled from the systemic vascular resistance. If, for example, MAP is mildly reduced due to a large decrease in cardiac output, cerebral vascular resistance would have to decrease as the rest of the body is vasoconstricted.

Anesthesia and critical care may entail large perturbations in both blood pressure and cardiac output, but the overriding treatment goal regardless of clinical condition is to maintain adequate organ perfusion and oxygenation. Although there has recently been some emphasis on the importance of cardiac output (Meng et al. 2015), the focus in the literature is still on MAP presented as the main determinant of an autoregulated cerebral blood flow, albeit with varying lower limits (Brady et al. 2020). Such a system may seem to disregard the complex interplay of the different components of the circulatory system. All organs seem to display some degree of (auto)regulation (Meng et al. 2019), and a reduction in CBF during reduced cardiac output has been found in several studies (Ogoh et al. 2005, 2015; Brown et al. 2003; Ogawa et al. 2007; Bronzwaer et al. 2017). CBF may, however, be on the top of the “regulatory hierarchy”, which is consistent with CBF constituting a higher proportion of cardiac output as cardiac output is reduced (Neumann et al. 2019). We found the estimated relative reduction in MCAV to be only approximately 25% of the reduction in cardiac output, consistent with the brain being given priority. Thus, the textbook presentation of cerebral autoregulation is at least imprecise (Willie et al. 2014), and the notion of a picture-perfect autoregulatory plateau has largely been abandoned. A more complex interplay where different autoregulatory plateaus exist for different levels of cardiac output has been proposed (Meng et al. 2015). Our findings indicate that CBF takes some part in the reduction in global blood flow during hypovolemia, in accordance with such a view, however with a more pressure passive response to MAP. Importantly, regulation of CBF may be influenced by different disease processes and anesthesia. However, the present results fit well with the finding that both the vasopressor phenylephrine and inotrope dobutamine increased graft flow during cerebral bypass surgery (Akkermans et al. 2020).

We have searched for upper and lower limits of autoregulation in our data with polynomial regression models but found the linear relationship to describe the data best. However, although we found a linear relationship between MAP and MCAV, this does not necessitate a totally pressure passive relation, as there may exist “relative autoregulation”. Our estimated relative increase in MCAV was only approximately 50% of the increase in MAP, which implies some degree of regulation in MCAV within our data. Furthermore, there may be steeper relationships (i.e. more pressure passive regions) outside our MAP observations. Some studies have described quite narrow limits of autoregulation (Tan 2012), which could exist within our observed data, but the present study was not designed to look for these.

A statistically significant interaction effect between MAP and cardiac output was found for ICAV and ScO_2_, however not reaching statistical significance for MCAV. These interaction effects might reflect the redundancy in the regulatory mechanisms for CBF, as the effects of changes in MAP were larger as cardiac output was reduced, and vice versa.

### ETCO_2_

Arterial partial pressure of CO_2_ (PaCO_2_) has a strong influence on CBF (Willie et al. 2014). In the present study however, we measured but did not clamp ETCO_2_. In many studies, ETCO_2_ is used as a proxy for PaCO_2_ as the latter requires arterial catheterization and cannot be measured continuously. ETCO_2_ is typically reduced with LBNP-induced central hypovolemia, which is often interpreted to reflect a reduction in PaCO_2_. Changes in ETCO_2_ are, however, associated with changes in cardiac output (Joseph et al. 2003; Lakhal et al. 2017) (Appendix, Fig. C). When we entered ETCO_2_ as an explanatory variable in the multivariable analysis with MAP, cardiac output and MCAV, the effect of cardiac output was reduced (Appendix, Regression 7). This, along with the close association between cardiac output and ETCO_2_ (Appendix, Fig C) indicates statistical multicollinearity, and that ETCO_2_ is redundant in the model when cardiac output is already entered. This further indicates that, in our model, changes in ETCO_2_ exert little independent effect when changes in cardiac output have been accounted for.

At least three mechanisms could explain a reduction in ETCO_2_ during hypovolemia: (1) a reduction in PaCO_2_ due to hyperventilation and/or reduction in peripheral CO_2_-production, (2) reduced PaCO_2_ due to sequestering of CO_2_ in peripheral tissues (decreased wash-out) (Garnett et al. 1989), or (3) reduced pulmonary blood flow, increased dead-space ventilation and increased alveolo-arterial CO_2_-difference. In the third case, a reduction in ETCO_2_ does not need to be associated with a reduction in PaCO_2_. Several studies indicate that this mechanism partly explains the reduction in ETCO_2_ with experimental (Dubin et al. 2000) and clinical hypovolemia (Campion et al. 2019; Tyburski et al. 2003), questioning the assumption that ETCO_2_ can substitute PaCO_2_. This finding is supported by studies applying orthostatic stress, where both LBNP (Zhang and Levine 2007) and head-up-tilt (Immink et al. 2006; Serrador et al. 2006) induced greater reductions in ETCO_2_ than PaCO_2_. Also, while controlling for the effect of PaCO_2_, they found an effect of cardiac output on CBF. A recent study reported stable PaCO_2_ in the LBNP model (van Helmond et al. 2018). As in other studies, we found a reduction in ETCO_2_ with increasing hypovolemia. To explore possible mechanisms behind this reduction, we plotted ETCO_2_ and cardiac output over time at release of LBNP (Appendix, Fig. N). The figure shows that both cardiac output and ETCO_2_ increase within 5 s, which seems hard to reconcile with mechanism (1), as PaCO_2_-production would increase much more slowly, and also mechanism (2), as even the wash-out and transport of CO_2_ from peripheral tissues to the lungs would be expected to take more time than what was observed. Hence, the reduction in ETCO_2_ in our study seems to be, at least in part, caused by increased alveolar dead-space ventilation. The relationship between ETCO_2_ and PaCO_2_ is, however, complicated, and may differ between acute changes and steady-state conditions (Isserles and Breen 1991). It is therefore difficult to state if or to what extent PaCO_2_ is changed in the present study and it would also question the value of clamping ETCO_2_. Further studies on the relationship between ETCO_2_ and PaCO_2_ in the LBNP model are warranted.

### Methodological considerations

As with other studies using transcranial Doppler (TCD) ultrasound, we were not able to measure the diameter of the MCA. Although the diameter is often assumed not to change, it is important to be aware of the fact that small changes in diameter may lead to large changes in flow that we were not able to detect. However, previous studies indicate that the caliber of the MCA remains quite constant during LBNP (Serrador et al. 2000) and blood pressure changes (Giller et al. 1993). The fact that we found a similar effect of cardiac output on CBF when using different methods (i.e. TCD and NIRS) where NIRS is independent of vessel diameter, indicates that the results are valid.

There are also limitations to consider regarding the ICA diameter measurements. As our priority in this study was continuous measurements of blood velocity, we imaged the ICA longitudinally. Hence, in the measurements of diameter, small and undetectable deviations from center may have led to foreshortening, and these results should therefore be interpreted with caution. Imaging the vessel in short axis would have allowed for more reliable measures of diameter but was not compatible with continuous measurements of blood velocity. ICA diameter could not be measured reliably in two subjects. For these reasons, we believe that the ICA diameters and derived blood flow values should be interpreted with caution.

Velocity–time integrals were calculated from the Doppler waveforms using automated software to reduce the risk of bias. These calculations were manually checked and corrected if obviously incorrect, before being accepted into the dataset. All Doppler measurements were performed by the same operators. Precision was calculated at LBNP 0 from the subjects starting without IHG, *i.e.* half the subjects.

ScO_2_ measured with NIRS is not a direct measure of CBF and have been shown to be contaminated by extracranial tissues (Davie and Grocott 2012). However, the method has gained popularity in clinical situations where cerebral perfusion may be compromised, and has been suggested as an alternative to transcranial Doppler (Rivera-Lara et al. 2017). We have previously demonstrated that changes in ScO_2_ seem to reflect changes in cerebral blood flow in the LBNP model (Hisdal et al. 2019). Although extracranial contamination probably affects the results of ScO_2_ in the present study, they are largely compatible with the results of MCAV and ICAV.

We did not study dynamic CA as we calculated mean values for periods of 30 s for each variable. Traditionally there has been made a distinction between studies on dynamic and static CA. However, this may only reflect the methodology used as there is little evidence for these being different physiological entities (Willie et al. 2014). Also, averaging values over a period of 15–30 s to eliminate the fluctuations caused by respiration is common (Kaur et al. 2018; Serrador et al. 2006; Ogoh et al. 2015; Washio et al. 2018; Tymko et al. 2016).

As seen in Figs. 3a, b, the relationship between MAP and MCAV, and cardiac output and MCAV, varied between subjects. To account for this, we also ran the analyses for MCAV with a random slope (random intercept—random slope model) to account for this difference between subjects. As the results were similar and did not lead to other conclusions, we present the results from our more parsimonious random intercept model.

It would be of potential value to study the isolated effects of changes in MAP and cardiac output at different fixed levels of the other, but we believe this was not feasible in the current model due to the integrated and dynamic nature of the cardiovascular response to the applied stimuli. Although LBNP mainly affects and reduces cardiac output, small changes in MAP may be seen. Conversely, IHG mainly increases MAP but may also slightly increases cardiac output (Stens et al. 2020). The regression models therefore attempt to describe the effect of one variable, controlling for the other. Nonetheless, our conclusions are restricted to the range of observed values during increased MAP and reduced cardiac output. Importantly, the cerebrovasculature seems to buffer increases in MAP better than reductions (Numan et al. 2014).

In conclusion, in a model of simultaneously increased MAP and reduced cardiac output in healthy volunteers, we found independent effects of these variables on MCAV, ICAV and ScO_2_. These findings support the notion that cardiac output plays a role in CBF regulation, and that there is interplay between MAP and cardiac output.

## Supplementary Information

Below is the link to the electronic supplementary material.

Supplementary file1 (DOCX 1741 kb)

## Data Availability

The code used to analyze the data from the current study is available from the corresponding author on reasonable request.

## Abbreviations

CA: Cerebral autoregulation
CBF: Cerebral blood flow
ETCO_2_: End-tidal carbon dioxide
HR: Heart rate
ICA: Internal carotid artery
ICAV: Internal carotid artery velocity
IHG: Isometric hand grip
LBNP: Lower body negative pressure
MAP: Mean arterial pressure
MCA: Middle cerebral artery
MCAV: Middle cerebral artery velocity
NIRS: Near-infrared spectroscopy
PaCO_2_: Partial pressure of carbon dioxide
ScO_2_: Cerebral oxygen saturation
SD: Standard deviation
SVR: Systemic vascular resistance
TCD: Transcranial Doppler

## Declarations

### Availability of data and material

The dataset generated during the current study is available from the corresponding author on reasonable request.
