# Supplementary material for: Cerebral blood flow velocity during simultaneous changes in mean arterial pressure and cardiac output in healthy volunteers
Source: Eur J Appl Physiol. 2021 Apr 22;121(8):2207–17. doi: 10.1007/s00421-021-04693-6 (PMC8260418; doi:10.1007/s00421-021-04693-6)
Supplement: Supplementary file 1 — Supplementary file1 (DOCX 1741 kb) [file 421_2021_4693_MOESM1_ESM.docx]

**Appendix**

All regressions are performed in a linear mixed model with a random intercept, with subjects as a random effect, if not noted otherwise.

**Abbreviations:**

| MCAV | Middle cerebral artery velocity |
| --- | --- |
| ICAV | Internal carotid artery velocity |
| MAP | Mean arterial pressure |
| ETCO_2_ | End-tidal carbon dioxide |
| ScO_2_ | Cerebral oxygen saturation |

# Regression 1, MCAV ~ MAP

**Dependent variable**

*MCAV (cm/s)*

**Explanatory variable**

*MAP (10 mmHg)*

|  | Estimate | Std. error | 95% CI | P-value |
| --- | --- | --- | --- | --- |
| Intercept | 28.77 | 5.05 | 18.87 to 38.68 | <0.001 |
| MAP (10 mmHg) | 3.43 | 0.36 | 2.72 to 4.13 | <0.001 |

# Regression 2, MCAV ~ cardiac output

**Dependent variable**

*MCAV (cm/s)*

**Explanatory variable**

*Cardiac output (L/min)*

|  | Estimate | Std. error | 95% CI | P-value |
| --- | --- | --- | --- | --- |
| Intercept | 46.22 | 4.15 | 38.09 to 54.36 | <0.001 |
| Cardiac output (L/min) | 3.69 | 0.34 | 3.02 to 4.36 | <0.001 |
|  |  |  |  |  |

# Regression 3, MCAV ~ MAP + cardiac output

**Dependent variable**

*MCAV (cm/s)*

**Explanatory variables**

*MAP (10 mmHg)*

*Cardiac output (L/min)*

|  | Estimate | Std. error | 95% CI | P-value |
| --- | --- | --- | --- | --- |
| Intercept | 16.45 | 5.07 | 6.51 to 26.39 | 0.0013 |
| MAP (10 mmHg) | 3.11 | 0.31 | 2.51 to 3.71 | <0.001 |
| Cardiac output (L/min) | 3.41 | 0.30 | 2.82 to 4.00 | <0.001 |
|  |  |  |  |  |

#

# Regression 4, MCAV ~ MAP + LBNP (as factor)

**Dependent variable**

*MCAV (cm/s)*

**Explanatory variables**

*MAP (10 mmHg)*

*LBNP 20 (dummy variable)*

*LBNP 40 (dummy variable)*

*LBNP 60 (dummy variable)*

*LBNP 80 (dummy variable)*

|  | Estimate | Std. error | 95% CI | P-value |
| --- | --- | --- | --- | --- |
| Intercept | 38.69 | 4.53 | 29.82 to 47.57 | <0.001 |
| MAP (10 mmHg) | 2.89 | 0.28 | 2.35 to 3.44 | <0.001 |
| LBNP 20 | -3.24 | 0.65 | -4.51 to -1.97 | <0.001 |
| LBNP 40 | -6.25 | 0.65 | -7.52 to -4.99 | <0.001 |
| LBNP 60 | -9.44 | 0.73 | -10.86 to -8.01 | <0.001 |
| LBNP 80 | -14.40 | 1.41 | -17.16 to -11.65 | <0.001 |


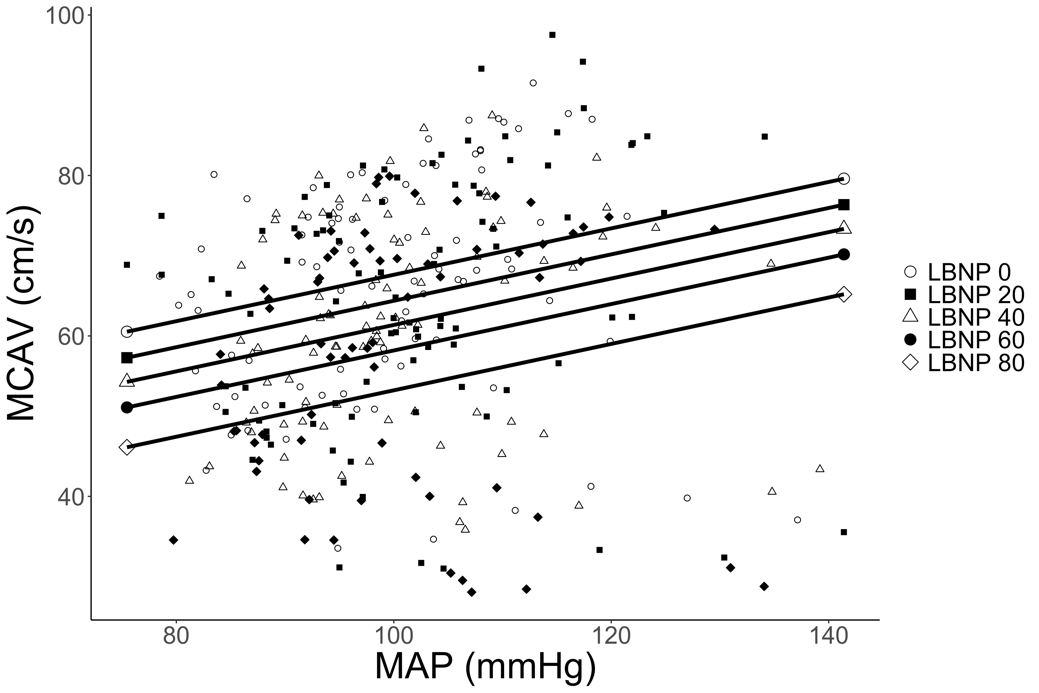


Fig. A. Middle cerebral artery velocity (MCAV) regressed on mean arterial pressure (MAP) with lower body negative pressure (LBNP)-level as a factor. The black lines represent the linear regression analyses for each LBNP-level. The symbols (i.e. open dots, squares, triangles etc.) represents data for each LBNP-level.

# Regression 5, MCAV ~ ETCO_2_

**Dependent variable**

*MCAV (cm/s)*

**Explanatory variable**

*ETCO_2_ (mmHg)*

|  | Estimate | Std. error | 95% CI | P-value |
| --- | --- | --- | --- | --- |
| Intercept | -15.28 | 7.76 | -30.50 to -0.07 | =0.0498 |
| ETCO_2_ (mmHg) | 2.12 | 0.18 | 1.76 to 2.49 | <0.001 |

**
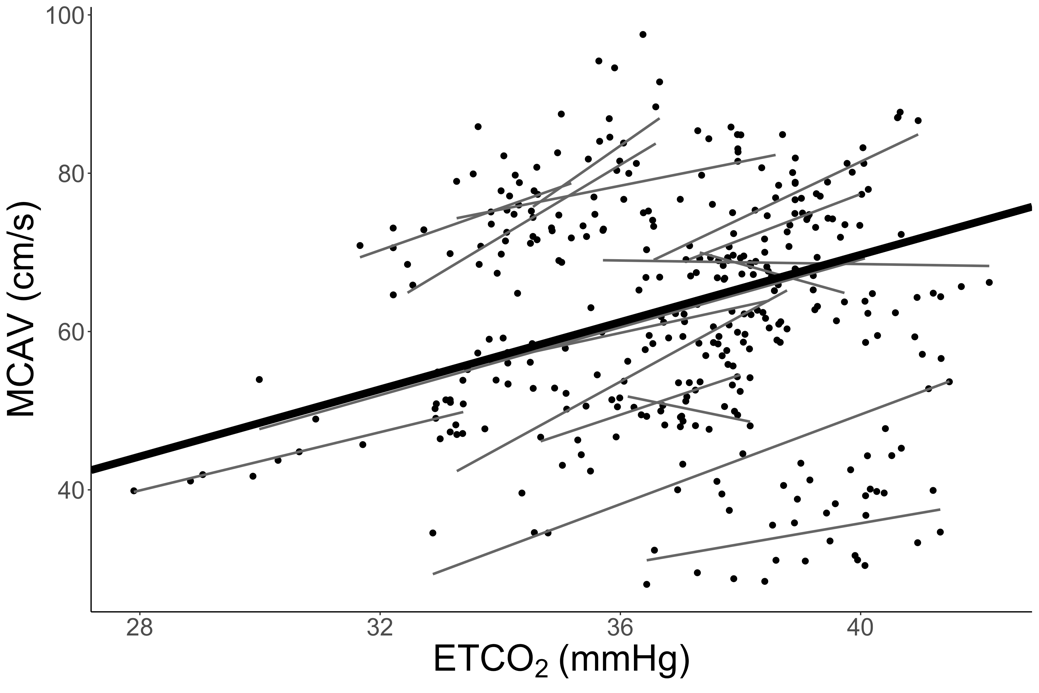
**

Fig. B. Middle cerebral artery velocity (MCAV) regressed on end-tidal carbon dioxide (ETCO_2_). The black line represents the mixed linear regression analysis, and black dots represent subject values with corresponding simple linear regressions represented as grey lines.

# Regression 6, cardiac output ~ ETCO_2_

**Dependent variable**

*Cardiac output (L/min)*

**Explanatory variable**

*ETCO_2_ (mmHg)*

|  | Estimate | Std. error | 95% CI | P-value |
| --- | --- | --- | --- | --- |
| Intercept | -6.63 | 0.90 | -8.40 to -4.86 | <0.001 |
| ETCO_2_ (mmHg) | 0.30 | 0.02 | 0.26 to 0.35 | <0.001 |

**
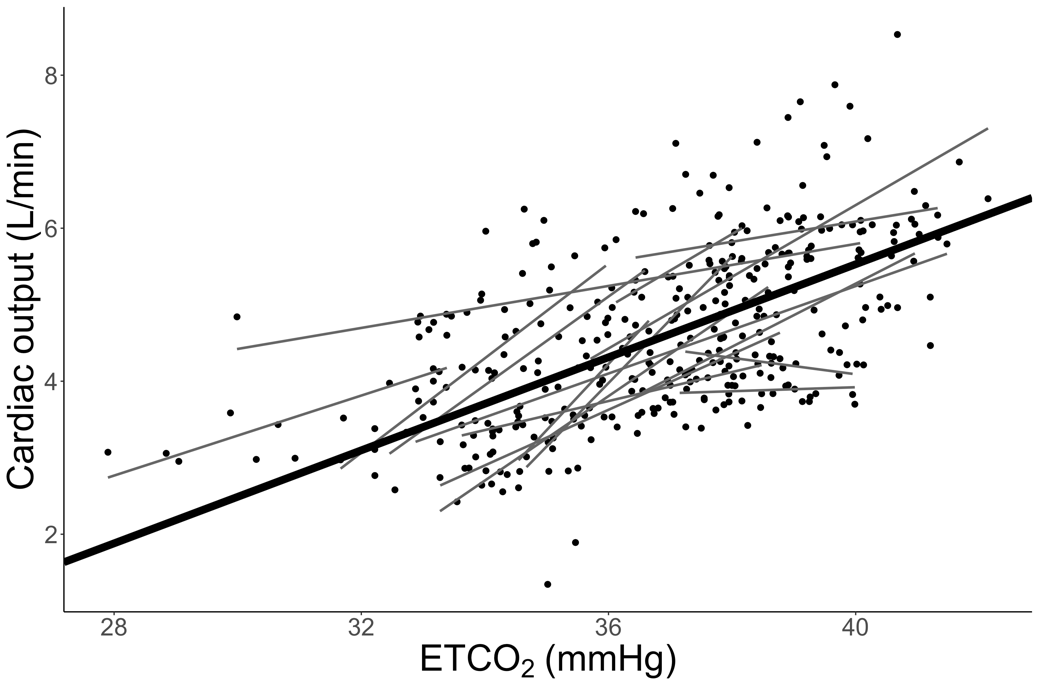
**

Fig. C. Cardiac output regressed on end-tidal carbon dioxide (ETCO_2_). The black line represents the mixed linear regression analysis, and black dots represent subject values with corresponding simple linear regressions represented as grey lines.

# Regression 7, MCAV ~ MAP + cardiac output + ETCO_2_

**Dependent variable**

*MCAV (cm/s)*

**Explanatory variables**

*MAP (10 mmHg)*

*Cardiac output (L/min)*

*ETCO_2_ (mmHg)*

|  | Estimate | Std. error | 95% CI | P-value |
| --- | --- | --- | --- | --- |
| Intercept | -31.08 | 7.68 | -46.14 to -16.02 | <0.001 |
| MAP (10 mmHg) | 3.14 | 0.28 | 2.59 to 3.68 | <0.001 |
| Cardiac output (L/min) | 1.88 | 0.33 | 1.23 to 2.54 | <0.001 |
| ETCO_2_ (mmHg) | 1.47 | 0.18 | 1.11 to 1.83 | <0.001 |

# Regression 8, ICAV ~ MAP

**Dependent variable**

*ICAV (cm/s)*

**Explanatory variable**

*MAP (10 mmHg)*

|  | Estimate | Std. error | 95% CI | P-value |
| --- | --- | --- | --- | --- |
| Intercept | 40.04 | 3.72 | 32.75 to 47.33 | <0.001 |
| MAP (10 mmHg) | 0.88 | 0.30 | 0.29 to 1.46 | 0.0037 |


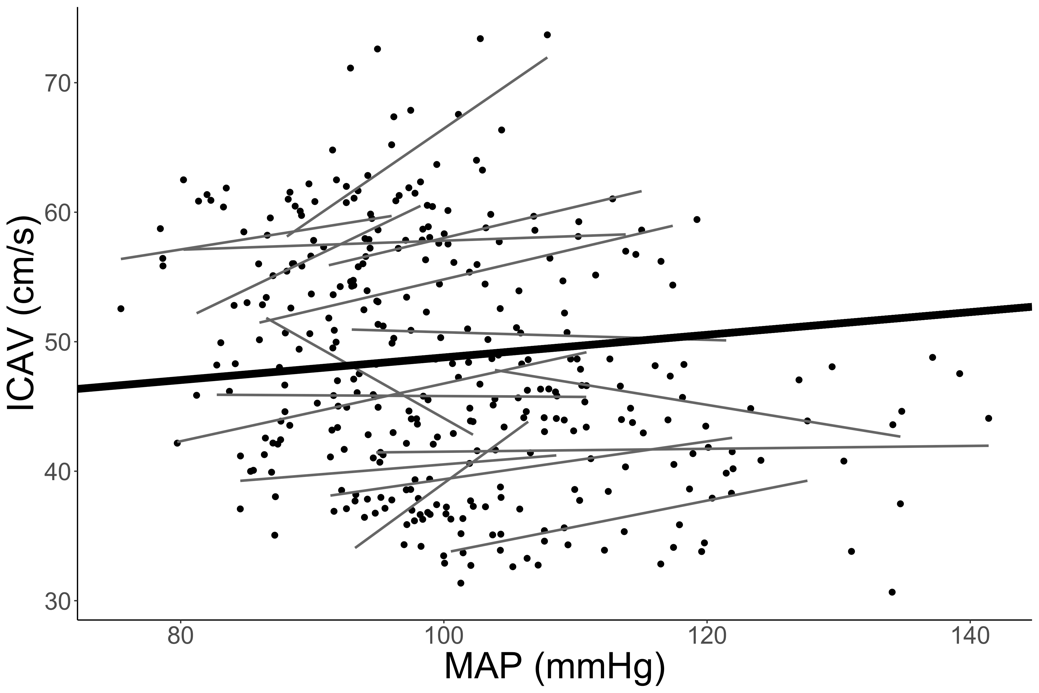


Fig. D. Internal carotid artery velocity (ICAV) regressed on mean arterial pressure (MAP). The black line represents the mixed linear regression analysis, and black dots represent subject values with corresponding simple linear regressions represented as grey lines.

# Regression 9, ICAV ~ cardiac output

**Dependent variable**

*ICAV (cm/s)*

**Explanatory variable**

*Cardiac output (L/min)*

|  | Estimate | Std. error | 95% CI | P-value |
| --- | --- | --- | --- | --- |
| Intercept | 45.06 | 2.58 | 40.00 to 50.11 | <0.001 |
| Cardiac output (L/min) | 0.83 | 0.32 | 0.21 to 1.44 | 0.0091 |


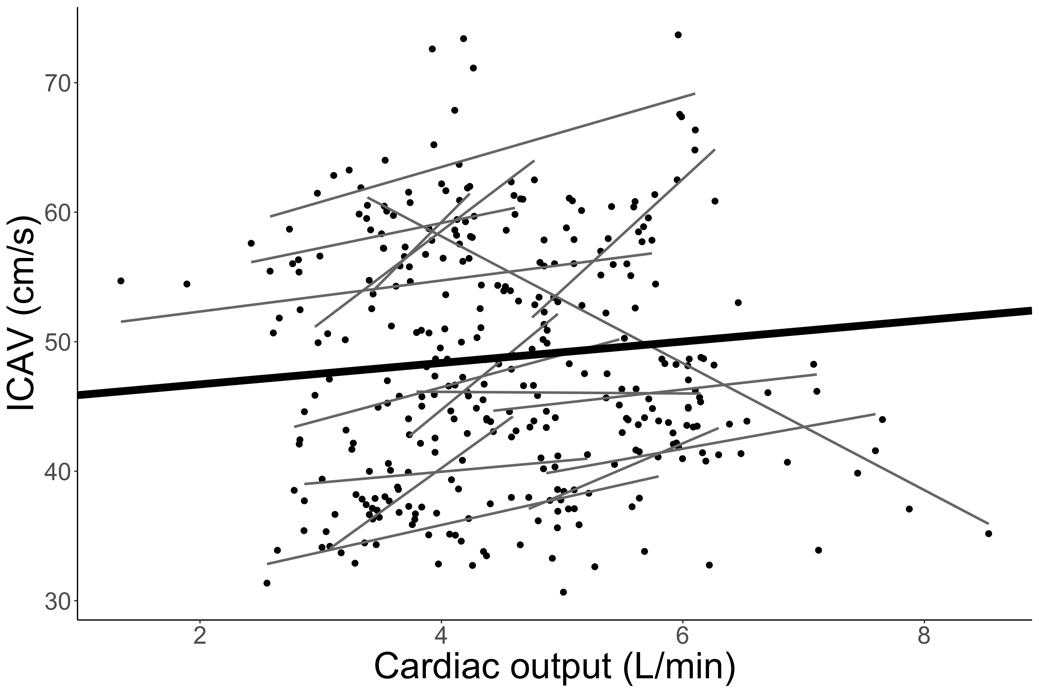


Fig. E. Internal carotid artery velocity (ICAV) regressed on cardiac output. The black line represents the mixed linear regression analysis, and black dots represent subject values with corresponding simple linear regressions represented as grey lines.

# Regression 10, ICAV ~ MAP * cardiac output

**Dependent variable**

*ICAV (cm/s)*

**Explanatory variables**

*MAP (10 mmHg)*

*Cardiac output (L/min)*

*MAP* × *Cardiac output*

|  | Estimate | Std. error | 95% CI | P-value |
| --- | --- | --- | --- | --- |
| Intercept | -11.28 | 11.73 | -34.26 to 11.70 | 0.337 |
| MAP (10 mmHg) | 5.67 | 1.15 | 3.43 to 7.92 | <0.001 |
| Cardiac output (L/min) | 10.71 | 2.29 | 6.21 to 15.20 | <0.001 |
| MAP × Cardiac output | -0.99 | 0.23 | -1.43 to -0.55 | <0.001 |

***Estimated effects of MAP at different percentiles of cardiac output***

|  | Cardiac output 25^th^ percentile (3.7 L/min) | Cardiac output 50^th^ percentile (4.5 L/min) | Cardiac output 75^th^ percentile (5.5 L/min) |
| --- | --- | --- | --- |
| MAP (10 mmHg) | 2.02 | 1.23 | 0.24 |

***Estimated effects of cardiac output at different percentiles of MAP***

|  | MAP 25^th^ percentile  (93 mmHg) | MAP 50^th^ percentile  (99 mmHg) | MAP 75^th^ percentile  (107 mmHg) |
| --- | --- | --- | --- |
| Cardiac output (L/min) | 1.51 | 0.92 | 0.13 |

# Regression 11, MCAV ~ ICAV

**Dependent variable**

*MCAV (cm/s)*

**Explanatory variable**

*ICAV (cm/s)*

|  | Estimate | Std. error | 95% CI | P-value |
| --- | --- | --- | --- | --- |
| Intercept | 30.33 | 4.50 | 21.51 to 39.15 | <0.001 |
| ICAV (cm/s) | 0.67 | 0.07 | 0.54 to 0.80 | <0.001 |


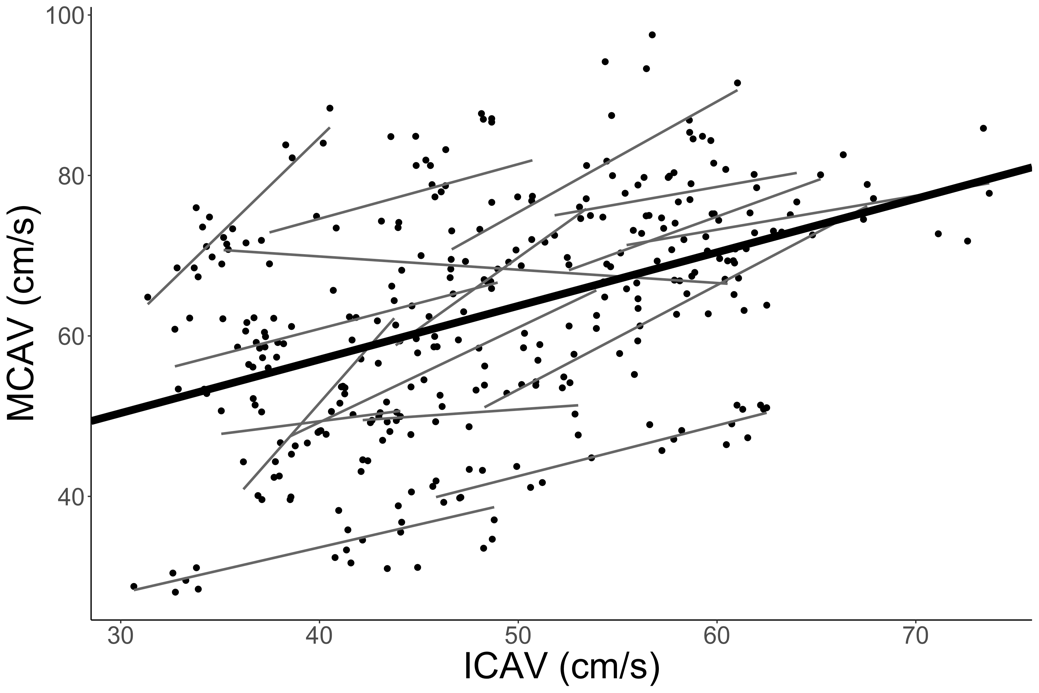


Fig. F. Middle cerebral artery velocity (MCAV) regressed on internal carotid artery velocity (ICAV). The black line represents the mixed linear regression analysis, and black dots represent subject values with corresponding simple linear regressions represented as grey lines.

# Regression 12, ICA blood flow ~ MAP

**Dependent variable**

*ICA blood flow (mL/min)*

**Explanatory variable**

*MAP (10 mmHg)*

|  | Estimate | Std. error | 95% CI | P-value |
| --- | --- | --- | --- | --- |
| Intercept | 221.57 | 38.42 | 146.27 to 296.88 | <0.001 |
| MAP (10 mmHg) | 5.83 | 6.68 | -0.71 to 12.37 | 0.0818 |


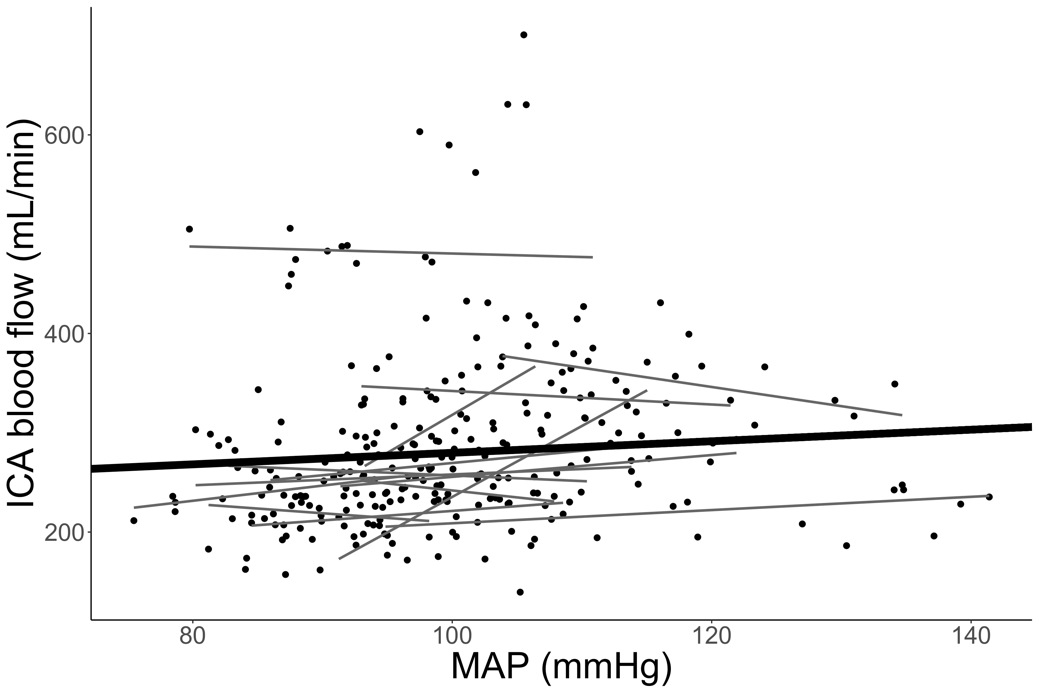


Fig. G. Internal carotid artery (ICA) blood flow regressed on mean arterial pressure (MAP). The black line represents the mixed linear regression analysis, and black dots represent subject values with corresponding simple linear regressions represented as grey lines.

# Regression 13, ICA blood flow ~ cardiac output

**Dependent variable**

*ICA blood flow (mL/min)*

**Explanatory variable**

*Cardiac output (L/min)*

|  | Estimate | Std. error | 95% CI | P-value |
| --- | --- | --- | --- | --- |
| Intercept | 224.47 | 26.57 | 172.39 to 276.56 | <0.001 |
| Cardiac output (L/min) | 11.94 | 3.77 | 4.54 to 19.33 | 0.0018 |


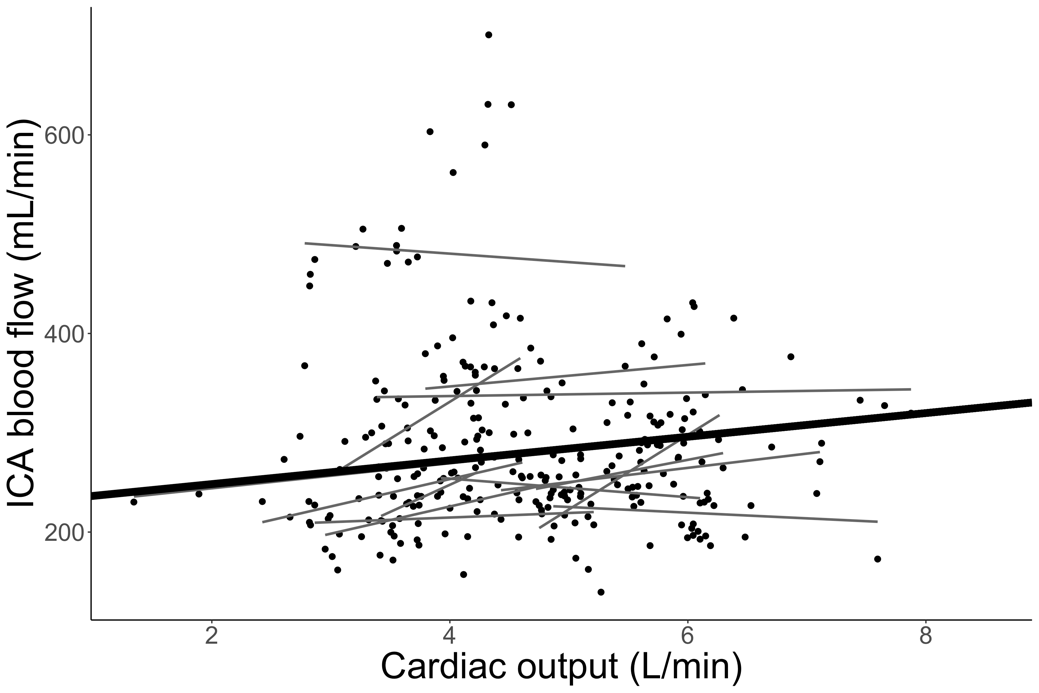


Fig. H. Internal carotid artery (ICA) blood flow regressed on cardiac output. The black line represents the mixed linear regression analysis, and black dots represent subject values with corresponding simple linear regressions represented as grey lines.

# Regression 14, ICA blood flow ~ MAP + cardiac output

**Dependent variable**

*ICA blood flow (mL/min)*

**Explanatory variables**

*MAP (10 mmHg)*

*Cardiac output (L/min)*

|  | Estimate | Std. error | 95% CI | P-value |
| --- | --- | --- | --- | --- |
| Intercept | 173.84 | 41.29 | 92.90 to 254.78 | <0.001 |
| MAP (10 mmHg) | 5.25 | 3.29 | -1.19 to 11.69 | 0.1115 |
| Cardiac output (L/min) | 11.60 | 3.77 | 4.21 to 18.99 | 0.0023 |

# Regression 15, ScO2 ~ MAP

**Dependent variable**

*ScO_2_ (%)*

**Explanatory variable**

*MAP (10 mmHg)*

|  | Estimate | Std.error | 95% CI | P-value |
| --- | --- | --- | --- | --- |
| Intercept | 68.04 | 1.98 | 64.16 to 71.92 | <0.001 |
| MAP (10 mmHg) | 1.02 | 0.13 | 0.76 to 1.27 | <0.001 |


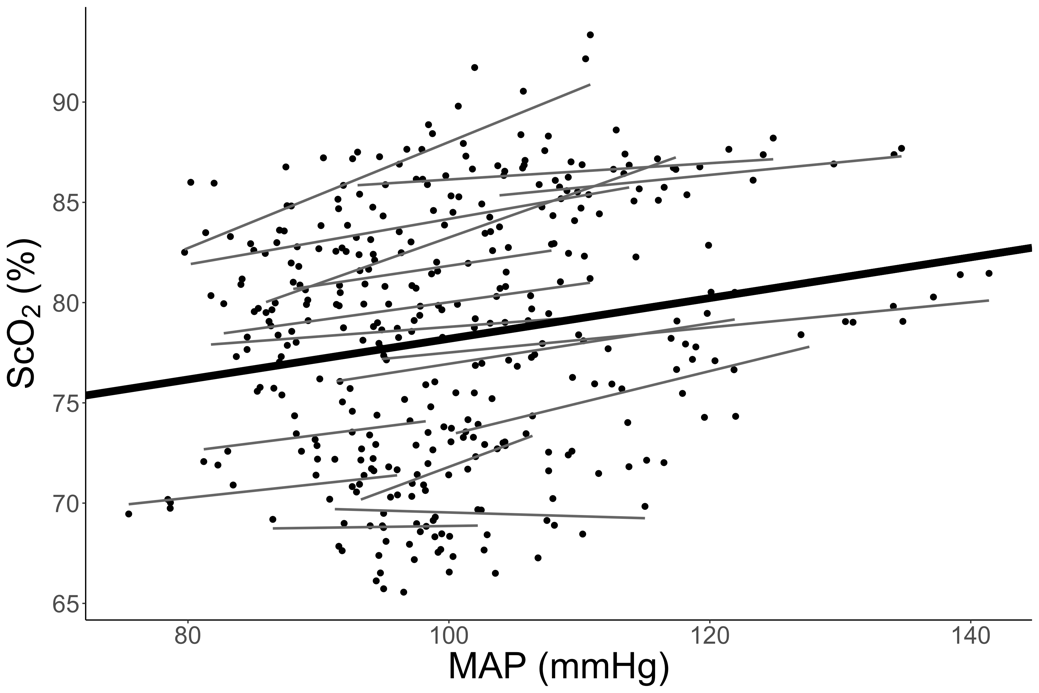


Fig. I. Cerebral oxygen saturation (ScO_2_) regressed on mean arterial pressure (MAP). The black line represents the mixed linear regression analysis, and black dots represent subject values with corresponding simple linear regressions represented as grey lines.

# Regression 16, ScO_2_ ~ cardiac output

**Dependent variable**

*ScO_2_ (%)*

**Explanatory variable**

*Cardiac output (L/min)*

|  | Estimate | Std.error | 95% CI | P-value |
| --- | --- | --- | --- | --- |
| Intercept | 75.27 | 1.60 | 72.14 to 78.41 | <0.001 |
| Cardiac output (L/min) | 0.64 | 0.13 | 0.34 to 0.90 | <0.001 |


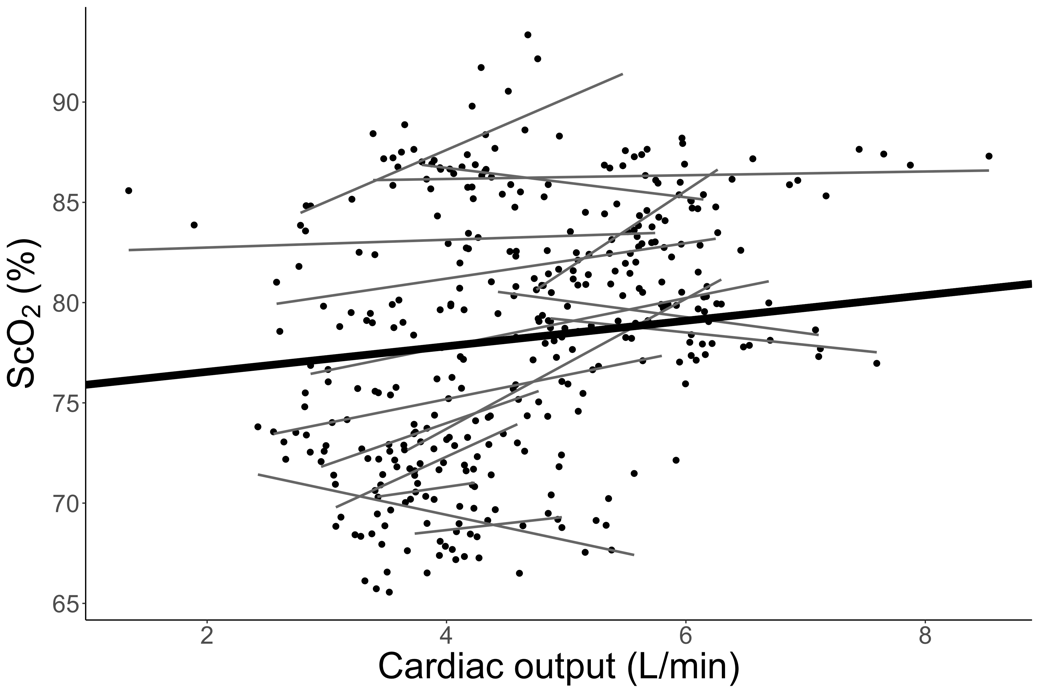


Fig. J. Cerebral oxygen saturation (ScO_2_) regressed on cardiac output. The black line represents the mixed linear regression analysis, and black dots represent subject values with corresponding simple linear regressions represented as grey lines.

# Regression 17, ScO2 ~ MAP * cardiac output

**Dependent variable**

*ScO_2_ (%)*

**Explanatory variables**

*MAP (10 mmHg)*

*Cardiac output (L/min)*

*MAP × Cardiac output*

|  | Estimate | Std.error | 95% CI | P-value |
| --- | --- | --- | --- | --- |
| Intercept | 50.77 | 5.19 | 40.59 to 60.94 | <0.001 |
| MAP (10 mmHg) | 2.51 | 0.50 | 1.53 to 3.49 | <0.001 |
| Cardiac output (L/min) | 3.67 | 0.98 | 1.75 to 5.59 | <0.001 |
| MAP × Cardiac output | -0.31 | 0.10 | -0.51 to -0.12 | =0.001 |

***Estimated effects of MAP at different percentiles of cardiac output***

|  | Cardiac output 25^th^ percentile (3.7 L/min) | Cardiac output 50^th^ percentile (4.5 L/min) | Cardiac output 75^th^ percentile (5.5 L/min) |
| --- | --- | --- | --- |
| MAP (10 mmHg) | 1.35 | 1.10 | 0.78 |

***Estimated effects of cardiac output at different percentiles of MAP***

|  | MAP 25^th^ percentile  (93 mmHg) | MAP 50^th^ percentile  (99 mmHg) | MAP 75^th^ percentile  (107 mmHg) |
| --- | --- | --- | --- |
| Cardiac output (L/min) | 0.75 | 0.56 | 0.31 |

# Regression 18, MCAV ~ ScO_2_

**Dependent variable**

*MCAV (cm/s)*

**Explanatory variable**

*ScO_2_ (%)*

|  | Estimate | Std. error | 95% CI | P-value |
| --- | --- | --- | --- | --- |
| Intercept | -58.11 | 11.01 | -79.69 to -36.53 | <0.001 |
| ScO_2_ (%) | 1.55 | 0.13 | 1.30 to 1.81 | <0.001 |


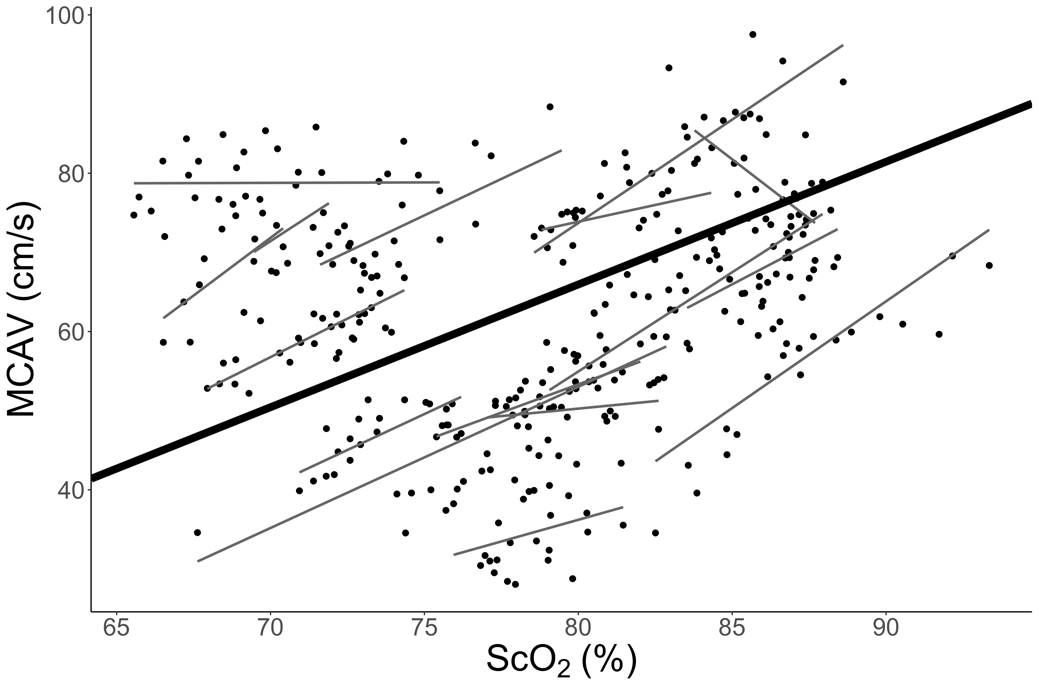


Fig. K. Middle cereral artery velocity (MCAV) regressed on cerebral oxygen saturation (ScO_2_). The black line represents the mixed linear regression analysis, and black dots represent subject values with corresponding simple linear regressions represented as grey lines.

# Regression 19, ICAV ~ ScO_2_

**Dependent variable**

*ICAV (cm/s)*

**Explanatory variable**

*ScO_2_ (%)*

|  | Estimate | Std. error | 95% CI | P-value |
| --- | --- | --- | --- | --- |
| Intercept | -1.12 | 8.89 | -18.54 to 16.31 | 0.9003 |
| ScO_2_ (%) | 0.64 | 0.12 | 0.42 to 0.85 | <0.001 |


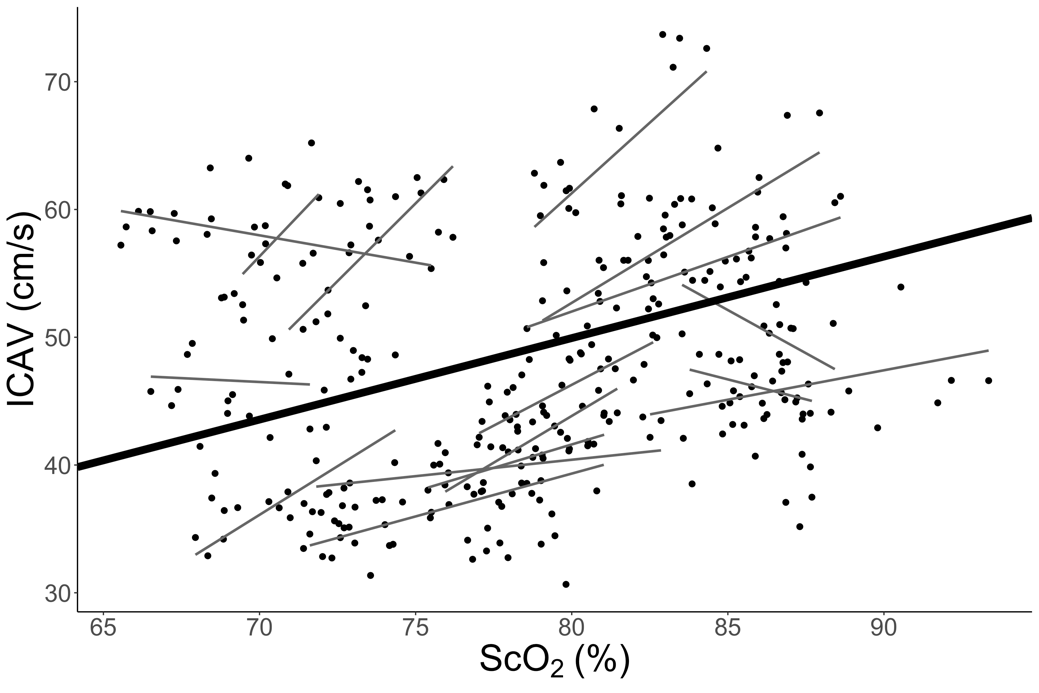


Fig. L. Internal carotid artery velocity (ICAV) regressed on cerebral oxygen saturation (ScO_2_). The black line represents the mixed linear regression analysis, and black dots represent subject values with corresponding simple linear regressions represented as grey lines.

# Regression 20, polynomial regression MCAV ~ MAP

To investigate the relationship between MCAV and MAP and to search for upper and lower limits of autoregultation within our data, we fitted polynomial models allowing for a sigmoid relationship. All data fitted in mixed linear models with subject as a random effect. Only the fixed effects are shown in the equations below.

Solid line: MCAV = β_0_ + β_1_ × MAP

Short dash: MCAV = β_0_ + β_1_ × MAP + β_2_ × (MAP)^3^

Long dash: β_0_ + β_1_ × MAP + β_2_ × (MAP)^2^ + β_3_ × (MAP)^3^


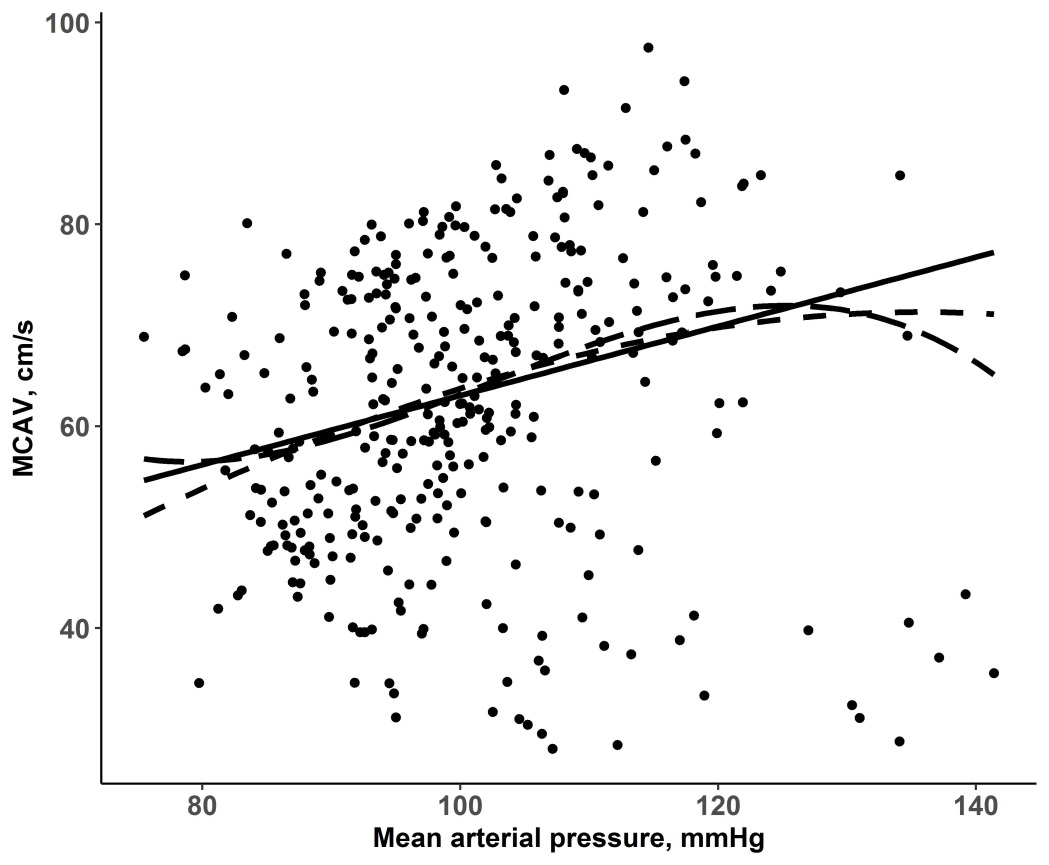


Fig. M. The model displaying a straight line (solid) had the best fit, as judged by the Akaike information criterion. Further, the models allowing for a curved relationship did not tend to curve as would be expected to model a lower and upper limit of autoregulation. We therefore believe the linear relationship, as assumed in our models, are adequate to fit our data.

#

# Fig. N, LBNP release


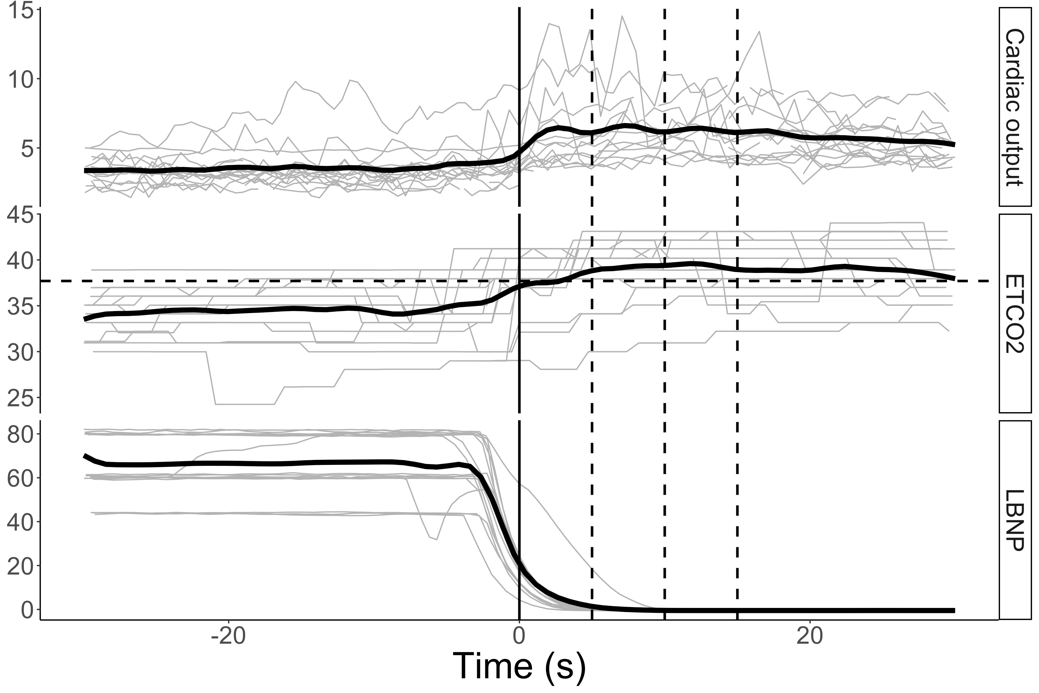


Fig. N. Cardiac output, end-tidal carbon dioxide (ETCO_2_) and lower body negative pressure (LBNP) at the release of LBNP (solid black vertical line) at the end of the experiment. Grey lines represent subjects and the solid line represent the average of all subjects. The dashed vertical lines represent time 5, 10 and 15 seconds after LBNP-release. The dashed horizontal line represents average ETCO_2_ at LBNP 0.
